# Flexible Smart Insole and Plantar Pressure Monitoring Using Screen-Printed Nanomaterials and Piezoresistive Sensors

**DOI:** 10.1021/acsami.5c08296

**Published:** 2025-07-29

**Authors:** Jaeho Lee, Jimin Lee, Yoon Jae Lee, Hodam Kim, Youngjin Kwon, Yunuo Huang, Matthew Kuczajda, Ira Soltis, Woon-Hong Yeo

**Affiliations:** † George W. Woodruff School of Mechanical Engineering, 1372 Georgia Institute of Technology, Atlanta, Georgia 30332, United States; ‡ Wearable Intelligent Systems and Healthcare (WISH) Center at the Institute for Matter and Systems, 1372 Georgia Institute of Technology, Atlanta, Georgia 30332, United States; § Parker H. Petit Institute for Bioengineering and Biosciences, 1372 Georgia Institute of Technology, Atlanta, Georgia 30332, United States; ∥ Department of Computer Science, Georgia State University, Atlanta, Georgia 30303, United States; ⊥ Department of Biomedical Engineering, Yonsei University, Wonju 26493, Republic of Korea; # School of Materials Science and Engineering, 1372 Georgia Institute of Technology, Atlanta, Georgia 30332, United States; ∇ School of Industrial Design, 1372 Georgia Institute of Technology, Atlanta, Georgia 30332, United States; ○ Wallace H. Coulter Department of Biomedical Engineering, Georgia Institute of Technology and Emory University School of Medicine, Atlanta, Georgia 30332, United States; ◆ Korea KIAT-Georgia Tech Semiconductor Electronics Center (K-GTSEC) at the Institute for Matter and Systems, 1372 Georgia Institute of Technology, Atlanta, Georgia 30332, United States

**Keywords:** wearable electronics, flexible smart insole, screen-printed nanomaterials, piezoresistive sensors, plantar pressure monitoring, gait analysis

## Abstract

Individuals experiencing gait dysfunction—such as the elderly, those with peripheral nervous system damage, or individuals with Parkinson’s disease—face a heightened risk of physical injury due to imbalanced weight distribution. Despite recent advancements in wearable movement trackers, there remains a significant need for a reliable long-term plantar pressure monitoring system. While some existing devices measure pressure characteristics, many are hindered by limitations in spatial resolution, sensitivity, and the presence of bulky peripherals. Here, we introduce a flexible smart insole system that integrates screen-printed nanomaterials to create a high-density piezoresistive sensor array, enabling accurate plantar pressure measurement during daily activities. To ensure scalable and cost-effective manufacturing, we utilize a screen-printing method to fabricate 173 carbon-based sensors directly onto a flexible insole circuit. The printed sensors demonstrate a remarkable sensitivity of −0.322 kPa^–1^, surpassing previous benchmarks. When combined with a wearable mobile communication circuit, this system offers a comprehensive analysis of the user’s plantar pressure distribution. Experimental studies conducted with human subjects showcase the smart insole’s real-time monitoring capabilities in common daily ambulation scenarios. The integration of high spatial resolution, exceptional sensitivity, and a fully mobile wearable system holds significant promise for enhancing outcomes across various applications, from healthcare to athletics.

## Graphical abstract

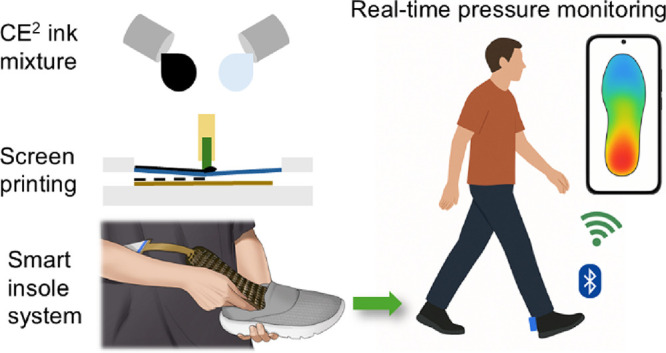

## 1 Introduction

Wearable electronics have emerged as a transformative family of tools for measuring key signals generated by our bodies. The comfort, portability, and seamless integration of “smart” patch-type devices,^1–4^ textiles and clothing,^5–7^ and wearable accessories^8,9^ make them an increasingly popular choice for unobtrusive monitoring of human physiological signals,^10–13^ chemical biomarkers,^14–16^ and movement.^17–19^ With such a variety of targets to measure and study, one signal that has been overlooked is plantar pressure. Wearable pressure sensors have tended to center around applications such as respiratory and cardiac monitoring,^20–22^ but plantar pressure is also a key metric that can be used to enhance healthcare outcomes. For example, previous studies have found that uneven plantar pressure distribution can be an effective diagnostic indicator for diabetic ulcers^23^ and rheumatoid arthritis,^24^ among various pathologies.^25^ Key characteristics of the gait cycle can also be derived from plantar pressure analysis.^26,27^ Features such as peak pressure, center of pressure (CoP), and gait phase timing are applicable to many clinical scenarios, especially characterization of neurological^28,29^ and musculoskeletal disorders.^30^ Plantar pressure sensors have previously been integrated into ground pads for gait analysis.^31–33^ However, these systems require on-site testing and are incapable of continuous monitoring during daily activities, limiting their use cases. As a result, recent research has shifted to the development of mobile sensing in the form of in-shoe pressure sensing insoles.

Several approaches have been evaluated for targeting plantar pressure measurement. Capacitive sensors offer good sensitivity but are limited by effective range and vulnerability to harsh conditions. Heavy loads and humid conditions due to sweat can degrade capacitive sensors’ long-term performance. Meanwhile, piezoelectric devices have attracted attention for their efficiency but suffer from off-axis interference and an inability to detect static forces. Less common approaches include optical, inductive, and air pressure-based systems. By contrast, piezoresistive sensing offers a useful combination of sensing performance, robustness, low cost, and ease of manufacturing.^34^

In terms of device architecture, many insoles place a small number of sensors in high-pressure zones around the forefoot and heel.^35,36^ Some examples even restrict the sensing area to a single unit, focusing on simple tasks such as step counting and thus suffering from lack of detail regarding pressure distribution across the whole foot.^37,38^ This tends to force interpolation of much of the distribution, leading to poor results in terms of real-time analysis of the pressure data. A high-density sensor array offers improvements over current insoles in spatial resolution^39^ thereby enabling enhanced analytical capabilities, such as precise CoP tracking and detailed gait analysis. Furthermore, many existing systems require bulky peripheral equipment for data acquisition and power delivery.^40,41^ Large, heavy ankle monitors are uncomfortable and impractical for continuous daily use. Superfluous wiring is also detrimental for user comfort and should be eliminated in the interest of practical wearability.

In this study, we present a smart insole that offers real-time monitoring and analysis of plantar pressure distribution. This device assesses plantar pressure with high spatial resolution by seamlessly integrating 173 piezoresistive sensors into an array with an accompanying data acquisition (DAQ) and Bluetooth Low Energy (BLE) communication circuit. The entire system is wearable and communicates wirelessly through the circuit, housed in a lightweight, low-profile case mounted on the heel. Even with its large coverage area, the insole retains excellent flexibility and durability using a custom carbon-epoxy-elastomer (CE^2^) ink mixture and screen-printing fabrication on a flexible printed circuit board (fPCB) substrate. By optimizing the balance of carbon, epoxy, and elastomer additive content in the ink, the sensors can be tuned to provide a highly sensitive response and working range matching the human body weight. With fast, accurate, real-time plantar pressure monitoring, this system will provide support to users in a variety of applications from clinical gait monitoring to sports performance analysis.

## 2 Results and Discussion

### 2.1 Design Overview and Operation of Smart Insole System

The overall structure and functionality of the smart insole are summarized in Figure 1. An important feature of the insole is its drop-in comfort and truly mobile capabilities. By using battery power and BLE technology, the system requires no wired connections. This avoids the bulky setup and stationary nature of traditional pressure measurement systems and provides everyday utility (Figure 1A). Real-time wireless data transfer from the insole to a laptop, tablet, or other portable device enables presentation of a range of beneficial health insights. A custom indexing algorithm scans the sensor array and provides updated pressure distribution information at a rate of 1 Hz. Heatmaps generated from sensor data provide intuitive graphical visualizations of plantar pressure distribution and could be used to indicate pressure hotspots or regions requiring ergonomic adjustments (Figure 1B). The high spatial resolution of the smart insole also has the potential to eventually support more advanced monitoring features. Examples include real-time visualization of the CoP trajectory, tracking regional pressure signals for detailed gait analysis, or a fall detection system. An fPCB was designed with 173 interdigitated electrodes (IDEs) connected into a grid by 14 vertical and 16 horizontal traces, all embedded within a US size 10 (men’s) insole platform (Figure 1C). Each IDE is 3.5 mm in diameter, with 15–18 mm between rows and 6–7 mm between columns. The electrodes and traces are composed of a 1 oz copper core with immersion gold treatment on the exposed electrode fingers. On top of this substrate, optimized CE^2^ ink mixture forms the sensing layer and 3 μm-thick parylene encapsulation completes the structure. This design enables a low overall thickness of 0.3 mm and a greater number of sensors than existing devices. The sensing platform also integrates a 4 × 4 cm^2^ DAQ circuit that clips directly to the user’s shoe. While other wearable devices may use bulky ankle monitors and straps, the minimal weight, compact circuit, and customized case create a comfortable and unobtrusive system for continuous real-time data collection. Additional information regarding the circuit designs and components is presented in Figures S1–S3.

**Figure 1:**
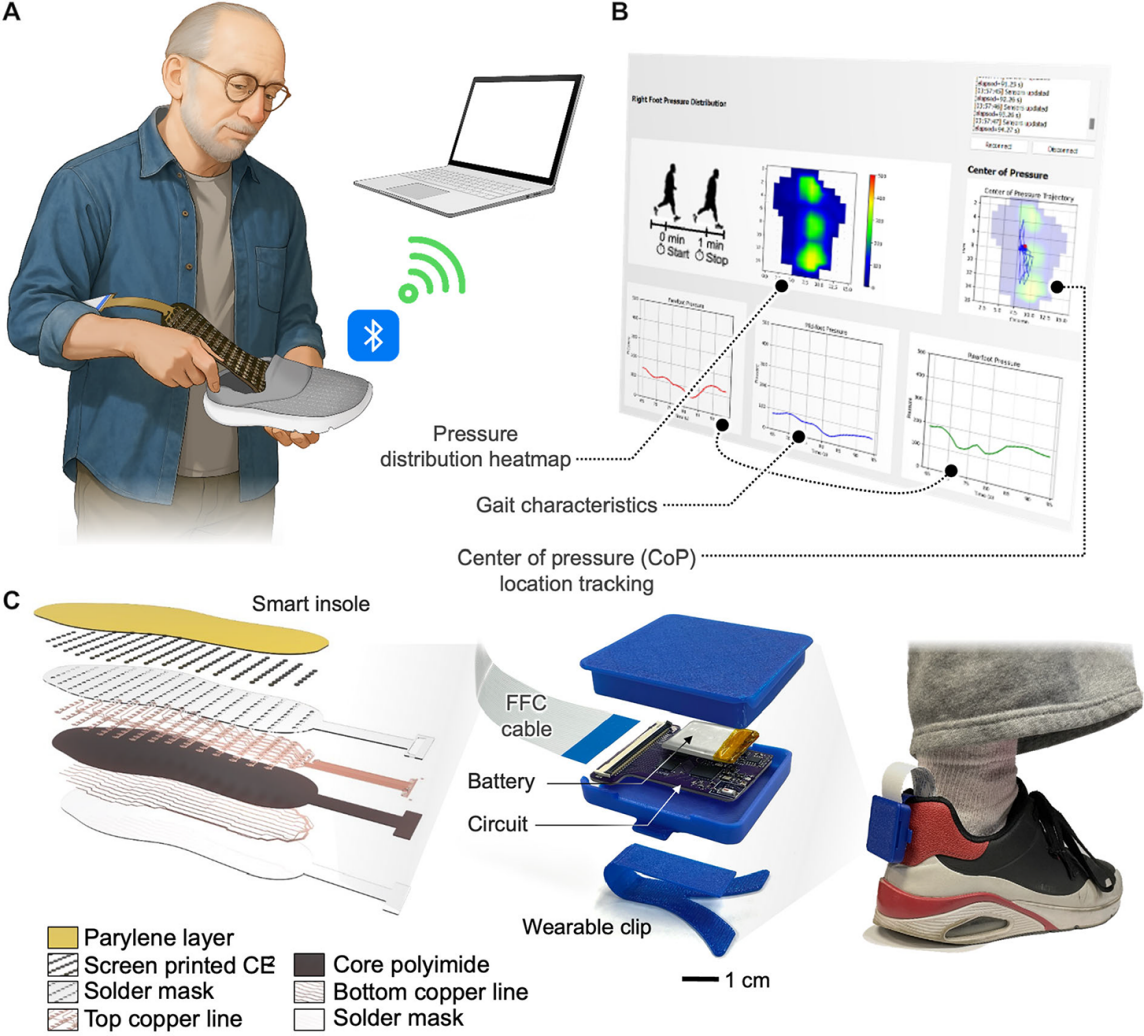
Overview of a flexible smart insole and plantar pressure monitoring system using screen-printed nanomaterials and piezoresistive sensors. (A) Illustration of the smart insole system in use. (B) Example of the user interface when connected to the insole system, showing pressure heatmap, gait characteristics, and CoP trajectory tracking. (C) Exploded view highlighting the screen-printed multilayered functional materials, integrated wireless electronics, and its low-profile form factor for user comfort.

### 2.2 Sensor Fabrication, Structure, and CE^2^ Optimization

A piezoresistive sensing approach was chosen for this smart insole due to its combination of great sensing performance, durability, and energy efficiency. Recent progress in pressure sensing technology supports the use of nanowires^42,43^ and nanoparticles,^44^ with some examples even being deployed specifically for insole applications.^45^ Thus, we selected a commercial carbon-epoxy ink as the base for the sensing layer and mixed it with an elastomer additive. Multiple studies have shown that such mixtures can introduce the percolation effect and improve sensitivity, providing good performance even in a smaller, thinner sensor.^46–49^ For this application, polydimethylsiloxane (PDMS) was used as the elastomer filler for the conductive carbon matrix. The modified ink mixture was applied to the IDEs using screen printing (Figure 2A; see the details of the screen-printing setup and process in Figures S4 and S5, respectively). This method is uniquely suited to rapidly creating thin layers over high-resolution, large-area patterns and takes very few processing steps. Screen printing is in widespread use in the electronics industry already, making the smart insole easily adapted to high-throughput manufacturing. Furthermore, advancements such as automatic screen printing can be combined with multilayer fPCBs to mitigate challenges during scale-up of the design to large-area applications. The structure of the individual pressure sensors is presented using optical microscope images and profilometer analysis (Figure 2B). Both the bare fPCB substrate and the complete insole with printed sensing layer were characterized. The overall thickness of the pristine IDE fingers is about 30 μm, while each layer of the printed CE^2^ adds approximately 20 μm to the overall height. This thin structure contributes to the flexibility and comfort of the insole during long-term use. The optical images show clearly defined layers of gold, ink, and polyimide encapsulation (Figure S6). In addition, the CE^2^ ink mixture was further studied and optimized for the application (see theoretical and physical changes in electromechanical properties as a function of carbon black-to-elastomer ratio in Figures S7 and S8; see the effect of the number of screen-printed layers on the surface profiles in Figure S9). Two aspects of the sensors were optimized for electrical conductivity (i.e., the lowest resistance): (1) the ratio of carbon-epoxy ink to elastomer and (2) the number of printed layers. Replicated IDE units were fabricated and analyzed using a 4-point probe and digital multimeter. Overall, a range of 5 ratios and 5 printed layer stacks were studied. Figure 2C shows the change in resistivity as the PDMS content is increased from 10% to 50% (wt %) of the CE^2^ ink mixture (*n* = 10). A smaller proportion of PDMS improves conductivity but causes cracks in the material, while higher ratios of elastomer significantly increase the resistivity. Screen printing allows for consistency in layer height and fine control over the total thickness of the deposited CE^2^ as shown in Figure 2D (*n* = 10). Finally, the results presented in Figure 2E demonstrate how the increased carbon content from additional printed layers provides more conductive pathways through the sensor, resulting in a large decrease in resistivity (*n* = 10). The results show that optimum characteristics can be achieved using a 70:30 ratio (wt %) of carbon-epoxy:PDMS and 5 printed layers, yielding a baseline resistance of ∼5 kΩ across each sensor while avoiding cracks.

**Figure 2:**
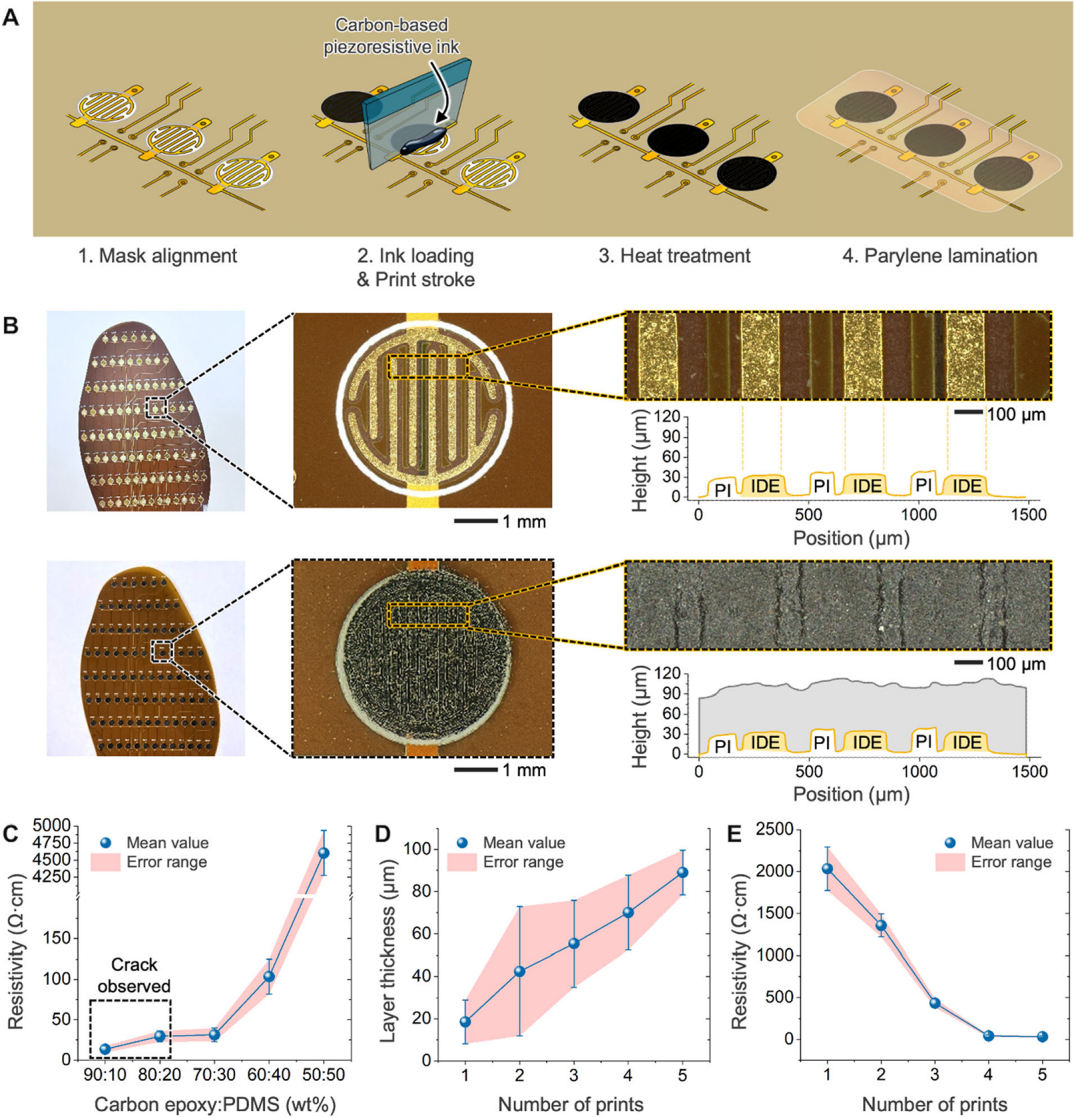
Optimization of CE^2^ ink mixture and fabrication parameters. (A) Illustration outlining primary fabrication steps of the piezoresistive sensor array. (B) Optical microscope images and surface characterization of the bare and printed IDEs. (C) Resistivity of printed CE^2^ when mixed at different ratios of carbon-epoxy ink and PDMS (*n* = 10). (D) Overall height of the sensor when printing 1–5 layers (*n* = 10). (E) Resistivity of printed CE^2^ when printing 1–5 layers (*n* = 10).

### 2.3 Material Characteristics and Sensing Performance

The optimized sensor was further characterized through a range of mechanical tests. A schematic of the general working principle shows how the percolation effect can be used to measure pressure changes in conductive matrices (Figure 3A). As the CE^2^ matrix experiences vertical loading, the carbon particles link to form increasing numbers of conductive pathways and yield a decrease in resistance. An equivalent circuit can be used to describe the components contributing to the measured resistance (Figure 3B). In the smart insole application, a good pressure sensor profile would show a stable baseline resistance with rapid changes corresponding to applied or removed loading forces (Figure 3C,D). Rapid response and recovery times of 72 ms (*n* = 1) are enabled through optimization of the sensor structure and materials (Figure 3D; see additional details in Figures S10 and S11). This enables prompt updates of the pressure distribution and other visualizations. A durability study was conducted (400 cycles; 90° maximum bending; see Figure S12 for test setup) to examine the mechanical and electrical stability of the sensors (*n* = 1). The resistance fluctuations remained stable over this cyclic period, with minimal baseline drift even under bending conditions more severe than typical in-shoe use (Figure 3E). It is worth noting that the CE^2^ sensors were encapsulated with parylene-C to mitigate environmental effects such as humidity. Additionally, given that the temperature and humidity inside a shoe typically remain within a relatively narrow range (28–34 °C, 60–65% RH), the impact of ambient variation on piezoresistive properties is considered negligible in practical use.^50^ No significant drift was observed in resistance during extended operation or cyclic testing (see Figure 3E), supporting the stability of the sensor under expected in-shoe conditions. Thus, we can be sure that the smart insole will hold up to difficult conditions in the dynamic high-pressure environment inside the shoe. This result also provides confidence that the sensor is operating purely as a pressure sensor, with minimal sensitivity to off-axis deformation. The sensor calibration results are shown in Figure 3F upon an applied pressure of 40 kPa (*n* = 1). When considering the full area of the smart insole, this range easily covers peak plantar pressures and is thus well suited to the target application.^39,51,52^ This ensures accurate data for a variety of users and allows for sensing in multiple conditions. For example, the wide sensing range would adapt well to activities of daily life, such as lifting heavy objects. The sensitivity was calculated from the calibration curve to be −0.322 kPa^–1^ (*n* = 1), demonstrating high resolution for detecting subtle pressure variations (Figure 3G). The competitive performance metrics achieved by our smart insole system make it an effective option for health monitoring applications, particularly for users prone to uneven gait or weight distribution issues.

**Figure 3:**
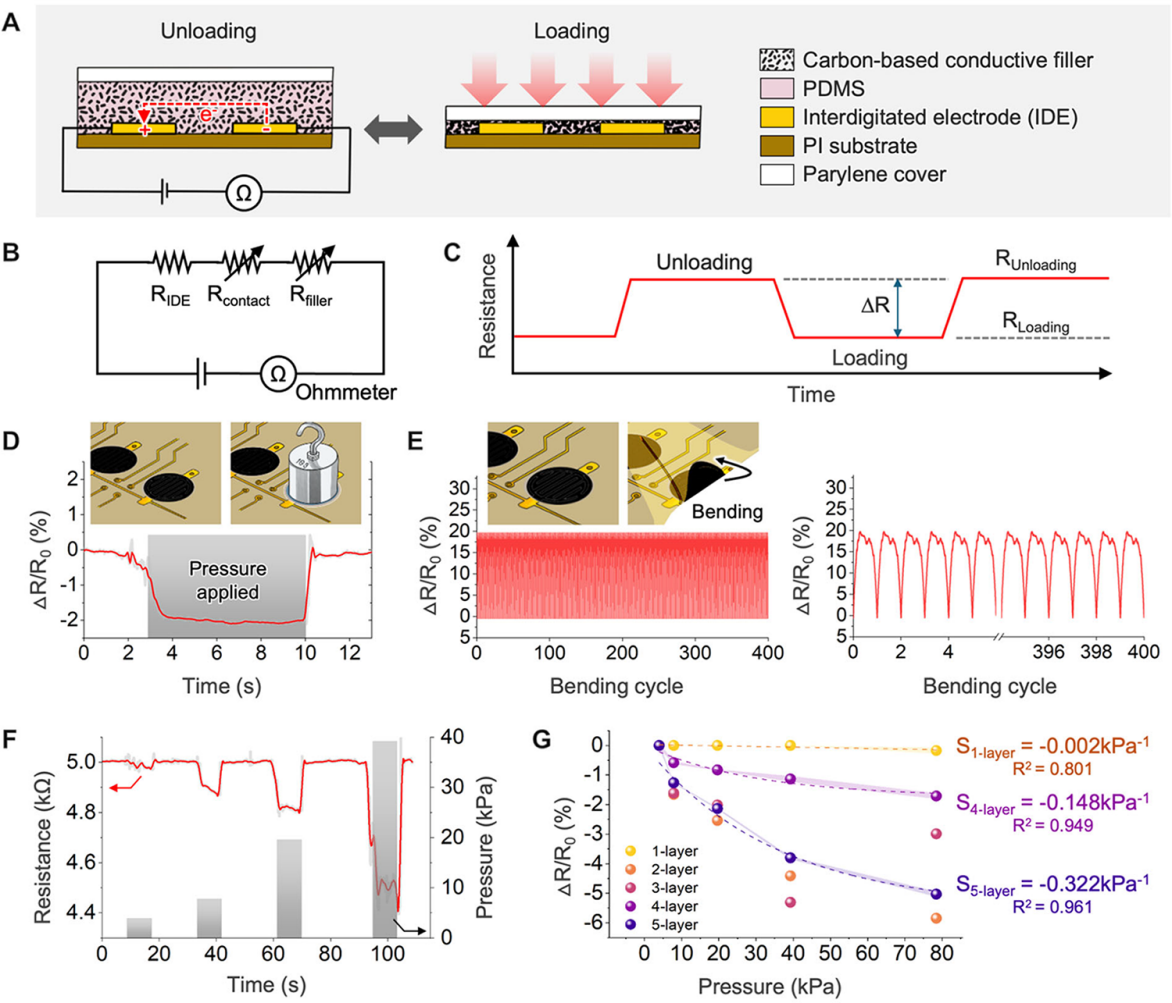
Mechanical and electrical characterization of pressure sensors. (A) Schematic illustration showing the piezoresistive working principle of the sensor. (B) Equivalent circuit for a single sensor array of the insole. (C) Theoretical response curve of a sensor during loading and unloading of applied pressure. (D) Single sensor (carbon-epoxy:PDMS = 70:30 (wt %)) response upon applied pressure (*n* = 1). (E) Durability test over 400 bending cycles at 90° (*n* = 1). (F) Calibration test results of a single sensor (carbon-epoxy:PDMS = 70:30 (wt %)) using four different calibration weights (*n* = 1). (G) Sensor sensitivity as a function of printed layer number (1–5 printed layers; *n* = 1).

### 2.4 System Validation with Human Subjects for Real-Time Plantar Pressure Monitoring

Multiple validation experiments were conducted to evaluate the smart insole’s functionality in real-time wireless monitoring scenarios. The insole connects to the wearable DAQ circuit via a single flat flexible connector (FFC) cable (Figure 4A). This minimalist wiring approach maximizes mobility, which is particularly beneficial during dynamic activities such as athletic training and rehabilitation exercises. In such applications, a conventional stationary pressure pad or bulkier insole system might hinder movement. The entire system maintains a lightweight (29 g) and low-profile design, enhancing user comfort during long-term daily use. It outperforms conventional systems that often rely on bulky ankle monitors or heavy enclosures. The flow of data from the sensor array to the user interface is detailed in Figure 4B. The real-time monitoring application (Figure 4C) provides comprehensive visualizations, including dynamic pressure distribution heatmaps. While a Windows PC was utilized as the primary display terminal for this study, the software architecture is easily adaptable to other platforms (e.g., smartphones and tablets). Figure 4D illustrates the spatial selectivity of the high-density smart insole sensor array. Various local pressure responses are shown via the application heatmap, demonstrating the system’s ability to track fine changes in the plantar pressure distribution throughout phases of the standard gait cycle. The high number of sensors and excellent spatial resolution also open the possibility of incorporating machine learning algorithms. This can be leveraged to provide features such as activity recognition using the large data sets generated during real-time monitoring. As shown in Table 1, our system’s capabilities match or exceed existing technologies when considering the overall package of spatial resolution, sensor performance and features, ease of fabrication, and wearability (note that sensitivity values are reported as magnitudes for ease of comparison). Overall, the unification of a high-density sensor array, low-profile wearable peripherals, and specialized software application unlocks a wide variety of functions that could prove beneficial in areas such as rehabilitation, gait monitoring, and sports performance analysis.

**Figure 4:**
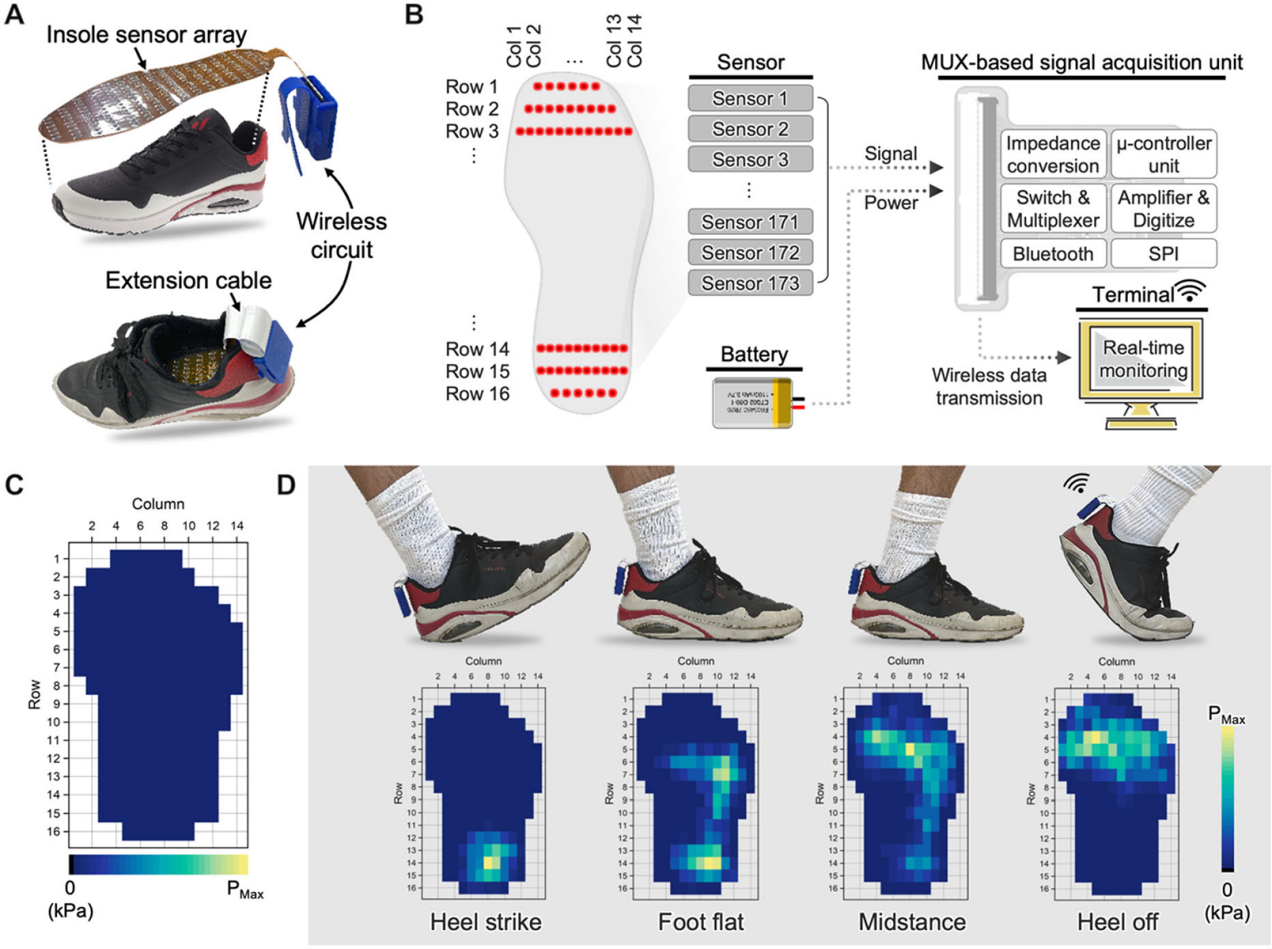
Real-time wireless pressure monitoring system. (A) Overview of the test setup for continuous pressure monitoring with the fabricated smart insole and integrated electronics. (B) Schematic of data acquisition and processing flow throughout the smart insole system. (C) Example of the heatmap as viewed in the user interface. (D) Validation of high spatial resolution with a human subject. Heatmaps show regional plantar responses during different phases of the gait cycle.

**Table 1:** Comparison between Our Work and Prior Research Measuring Plantar Pressure

refs	sensing modality	sensor count	real-time features	sensitivity (kPa^–1^)	response time (ms)	fabrication method	wireless capability
this work	piezoresistive	173	pressure heatmap	0.322	72	screen printing	yes
Chen et al.^53^	capacitive	7	pressure heatmap	0.26	15	drop casting	
Deng et al.^54^	piezoelectric	32	pressure heatmap	0.008	55	solution synthesis	
Guo et al.^37^	piezoelectric	1	pressure signal	0.017	290	solution synthesis electrospinning	yes
Nie et al.^55^	inductor-capacitor	8	pressure heatmap	0.19	100	screen printing, wet etching, laser machining	
Xu et al.^38^	triboelectric	1	pressure signal			solution synthesis, drop casting	
Tao et al.^56^	capacitive	24	pressure heatmap	0.012	142	polymer molding and casting, laser cutting,	yes
Beccatelli et al.^35^	piezoelectric	8	pressure heatmap	0.3		solution synthesis, molding	
Xiang et al.^57^	piezoresistive	32	pressure heatmap		140	leather processing, solution synthesis	
Li et al.^58^	piezoresistive	104	pressure heatmap, activity classification	0.01	82	fiber weaving, adhesives, laser cutting	yes
Sun et al.^59^	piezoresistive	7	gait recognition	0.0062–0.88		laser patterning and cutting, polymer casting, screen printing	yes
Wang et al.^60^	piezoresistive	22	pressure heatmap, activity classification	0.36		spin coating, immersion, photolithography, sputtering, laser cutting	yes

## 3 Conclusions

This article reports on a flexible smart insole system featuring screen-printed nanomaterials and a high-density piezoresistive sensor array that demonstrates exceptional sensitivity and range. The insole is designed with an extremely thin, flexible structure based on a robust substrate, ensuring facile construction. Through extensive optimization of the ink composition and screen-printing parameters, we have achieved an ideal balance between a large sensing area and effective individual sensor performance. Force testing reveals that our smart insole achieves a sensitivity of −0.322 kPa^–1^ and a response time of 72 ms, with a sensing range suitable for measuring typical human body weights. Demonstrations with human subjects capture the system’s spatial resolution and selectivity in various ambulatory scenarios. The insole leverages its high sensor density to differentiate phases of the gait cycle, providing accurate real-time plantar pressure mapping. To support long-term use, the sensor layer, encapsulated with parylene-C, provides environmental and mechanical protection, while a 2-point calibration routine ensures consistent sensor behavior over time. In the event of sensor degradation, the insole can be easily replaced due to the low-cost, scalable screen-printing process on flexible PCBs, making the system not only high-performing but also practical for commercial deployment. Collectively, the smart insole system presented here stands to benefit innovative technologies that depend on high spatial resolution and substantial data volumes, such as human-in-the-loop wearable robotics and continuous health monitoring.

## 4 Experimental Section

### 4.1 Materials

Carbon-epoxy ink (120–24–2K, Creative Materials Inc.) and polydimethylsiloxane (PDMS; Sylgard 184 silicone elastomer, DOW Inc.) were the primary components used for screen printing. Screen-printing equipment was sourced from Hary Manufacturing Inc. (Lebanon, NJ, USA). Both flexible (for insole substrate) and rigid (for DAQ circuit) PCBs were provided by OSH Park (Lake Oswego, OR, USA). Details of the PCB designs are outlined in Supporting Information (Figures S1–S3). Integrated circuit chips and passive components were acquired from DigiKey (Thief River Falls, MN, USA) and Mouser Electronics (Mansfield, TX, USA).

### 4.2 Sensor Fabrication

PDMS was prepared by combining the base and curing agent in a 10:1 weight ratio. The CE^2^ mixtures (∼15 g per batch) were prepared with varying weight ratios of carbon-epoxy ink to PDMS, ranging from 50:50 to 90:10, and homogenized using a centrifugal mixer (330-100 PRO, FlackTek) to ensure homogeneity. Before printing, the fPCB insole was cleaned with isopropanol, dried, and secured to an 8″ × 12″ glass plate using Kapton tape. The plate was then mounted onto the printer (MSP-485, Hary Manufacturing Inc.), where it held in position via vacuum suction. The integrated laser alignment system was utilized to precisely position the insole beneath the screen pattern. The ink mixture was manually loaded onto the screen and applied with a single squeegee stroke. The printed sensor array was dried in a fume hood for 10 min before undergoing thermal curing on a hot plate at 175 °C for 30 min. To optimize the thickness of the printed layer, the printing process was repeated up to 5 times, followed by additional thermal treatment. Finally, the printed sensors were encapsulated with a parylene-C film (see Figure S13), and a 32-pin FFC connector was manually soldered onto the heel-mounted terminal strip.

### 4.3 Characterization

The dimensions and surface morphology of the fPCB were analyzed using a profilometer (VK-X3000, Keyence) and field-emission scanning electron microscopy (FE-SEM, SU8230, Hitachi), respectively. The screen-printed layers were imaged using an optical microscope (VHX-7000, Keyence) to assess layer thickness and uniformity. Additionally, a four-point probe measurement system (SYS-301, Signatone) was used to determine the resistivity (Ω·cm) and sheet resistance (Ω/sq) under different printing conditions. For mechanical and electrical stability measurements, a motorized vertical test stand (ESM303, Mark-10) equipped with a force gauge (M5–5, Mark-10) was used in conjunction with an LCR meter (BK891, B&K Precision). All resistance values from sensor response testing were recorded using a digital multimeter (DMM7510, Keithley).

### 4.4 Data Circuit

The electronic subsystem of the smart insole was designed to support robust, real-time, high-resolution, impedance-based pressure sensing with wireless data transmission. Key components of the circuit design are described in Figure S3.

### 4.5 Sensing and Amplification

The insole’s DAQ circuit employs an operational amplifier (AD8606, Analog Devices) to amplify signals from multiple pressure sensors. An analog switch (ADG849, Analog Devices) routes sensor outputs to the impedance converter for calibration and measurement. The sensor array signals are interfaced through an FFC connector (XF3M(1)-3215-1B, Omron Electronics) to ensure robust connectivity. Stabilized capacitors are used to ensure a consistent power supply and reduce noise, thereby enhancing signal fidelity under dynamic conditions.

### 4.6 Multiplexing for Multisensor Inputs

Multiple pressure sensors, distributed across the forefoot, midfoot, and heel regions, capture spatial pressure variations in plantar pressure. Two analog multiplexers (MUX1 and MUX2; ADG1606, Analog Devices) sequentially route each sensor’s output to the impedance converter, allowing efficient multisensor data acquisition without adding circuit complexity.

### 4.7 Analog-to-Digital Conversion (ADC)

The sensor signals are digitized using an impedance converter (AD5933, Analog Devices), specifically chosen for precise impedance measurements across the sensor array. The ADC’s reference voltage is stabilized by a resistor (R80) to ensure consistent performance under varying operational conditions.

### 4.8 Microcontroller and Wireless Transmission System

An nRF52832 microcontroller (Nordic Semiconductor) manages sensor data acquisition and Bluetooth-based wireless transmission, allowing real-time data transfer to external devices. An L-C filter stabilizes the power to the microcontroller, supporting consistent functionality even during high data transfer intervals. Designed for power efficiency, the system operates on a 150 mAh lithium polymer (LiPo) battery, for prolonged use in everyday wearable applications.

### 4.9 Firmware and Software

The firmware implements a streamlined data acquisition and BLE communication protocol designed to interface with the AD5933 impedance measurement module. Utilizing embedded SoftDevice-based protocols, the firmware systematically controls multiple hardware components, including the multiplexers and impedance converter, to precisely capture signals from the sensor array. Communication between the microcontroller and AD5933 is efficiently managed via the Two-Wire Interface (TWI) protocol, allowing detailed configuration of operational parameters such as frequency sweep, gain adjustments, and output ranges. Optimized for minimal latency (<10 ms), the firmware ensures rapid initialization, precise calibration, and continuous real-time data acquisition. Additionally, the firmware incorporates comprehensive error handling, data integrity checks, and command processing routines to guarantee stable operation during prolonged use. The software component, developed in Python, interfaces with the smart insole system via BLE, supporting connections to laptops and desktop environments. A simple plug-and-play graphical user interface (GUI) enables real-time data collection, intuitive visualization, and analysis of sensor data. Incoming data streams are systematically parsed, thread programmed and managed through efficient memory structures such as circular buffers, facilitating rapid data visualization with multithreading. Real-time visualizations include dynamic heatmaps for spatial pressure distribution. Automated data storage mechanisms are integrated, enabling data from each measurement session to be saved in.csv format, supporting subsequent detailed analysis or potential integration into machine learning pipelines. These features provide immediate insights into gait dynamics and plantar pressure distribution, promoting extensive applicability in clinical and research contexts.

### 4.10 Human Subject Study

A few healthy subjects participated in the study. The experimental protocol (IRB2025-61) was approved by the Georgia Tech Institutional Review Board, ensuring compliance with ethical research standards. In accordance with ethical guidelines, all participants provided written informed consent before the study.

## Supplementary Material



## Data Availability

The data supporting the findings of this study are available from the corresponding author upon reasonable request.

## Notes

Insole substrate design; DAQ circuit layout; summary of IC components for 32-channel impedance DAQ circuit; screen-printing setup and process; cross-sectional view of the as-prepared sensor; relationship between sensor properties and carbon content; surface profiles of printed samples with varying CE^2^ ratios; surface profiles of printed samples with different numbers of printed layers; characterization of sensor response time under applied pressure; test setup for sensor calibration; test setup for durability study; Parylene-C coating setup (PDF)
