# Supplementary material for: Flexible Smart Insole and Plantar Pressure Monitoring Using Screen-Printed Nanomaterials and Piezoresistive Sensors
Source: ACS Appl Mater Interfaces. 2025 Jul 29;17(33):47153–61. doi: 10.1021/acsami.5c08296 (PMC12371697; doi:10.1021/acsami.5c08296)
Supplement: Supplementary file 1 [file am5c08296_si_001.pdf]

Supporting Information

Flexible Smart Insole and Plantar Pressure Monitoring Using Screen-Printed Nanomaterials and Piezoresistive Sensors

Jaeho Lee [a,b,c,†], Jimin Lee [a,b,†], Yoon Jae Lee [b,d], Hodam Kim [b,e], Youngjin Kwon [b,f], Yunuo Huang [b,g], Matthew Kuczajda [a,b], Ira Soltis [a,b], Woon-Hong Yeo [a,b,c,h,i,*]

[a] George W. Woodruff School of Mechanical Engineering, Georgia Institute of Technology, Atlanta, GA, 30332, USA
[b] Wearable Intelligent Systems and Healthcare (WISH) Center at the Institute for Matter and Systems, Georgia Institute of Technology, Atlanta, GA, 30332, USA
[c] Parker H. Petit Institute for Bioengineering and Biosciences, Georgia Institute of Technology, Atlanta, GA, 30332, USA
[d] Department of Computer Science, Georgia State University, Atlanta, GA, 30303, USA
[e] Department of Biomedical Engineering, Yonsei University, Wonju, 26493, Republic of Korea
[f] School of Materials Science and Engineering, Georgia Institute of Technology, Atlanta, GA, 30332, USA
[g] School of Industrial Design, Georgia Institute of Technology, Atlanta, GA, 30332, USA
[h] Wallace H. Coulter Department of Biomedical Engineering, Georgia Institute of Technology and Emory University School of Medicine, Atlanta, GA, 30332, USA
[i] Korea KIAT-Georgia Tech Semiconductor Electronics Center (K-GTSEC) at the Institute for Matter and Systems, Georgia Institute of Technology, Atlanta, GA, 30332, USA
† Jaeho Lee and Jimin Lee equally contributed to this work
* Corresponding author. E-mail address: whyeo@gatech.edu

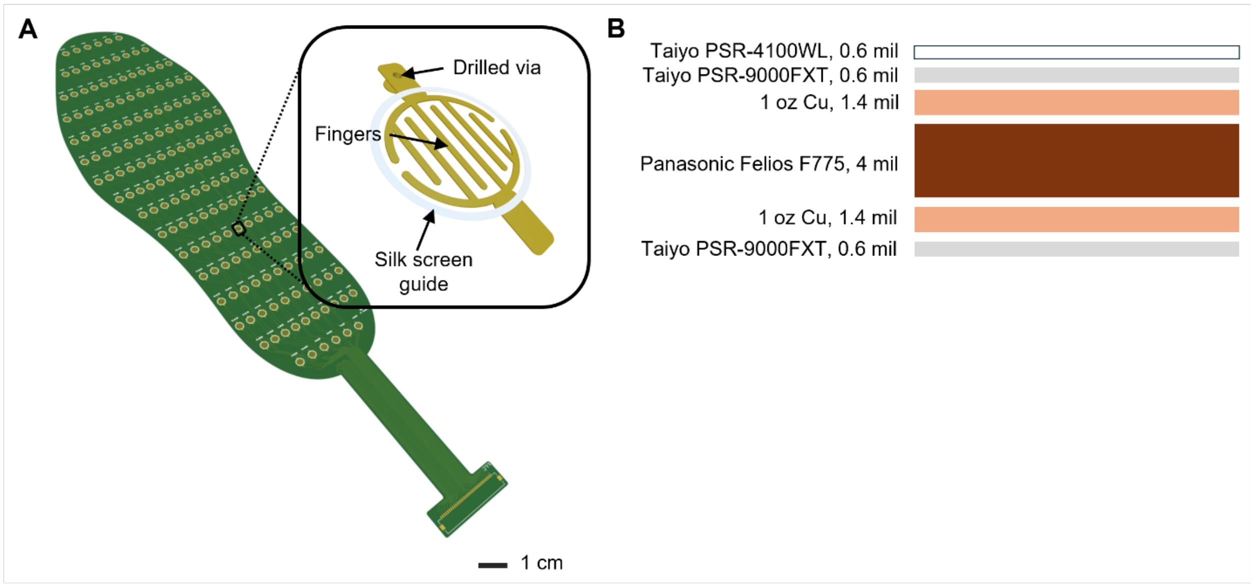

Figure S1. Insole substrate. (A) 3D model of fPCB design with detail of interdigitated electrode. The insole uses 6 mil (0.152 mm) traces and 10 mil (0.254 mm) vias. Each electrode finger is 7 mil (0.178 mm) in width. (B) Schematic of stackup layers comprising fPCB.

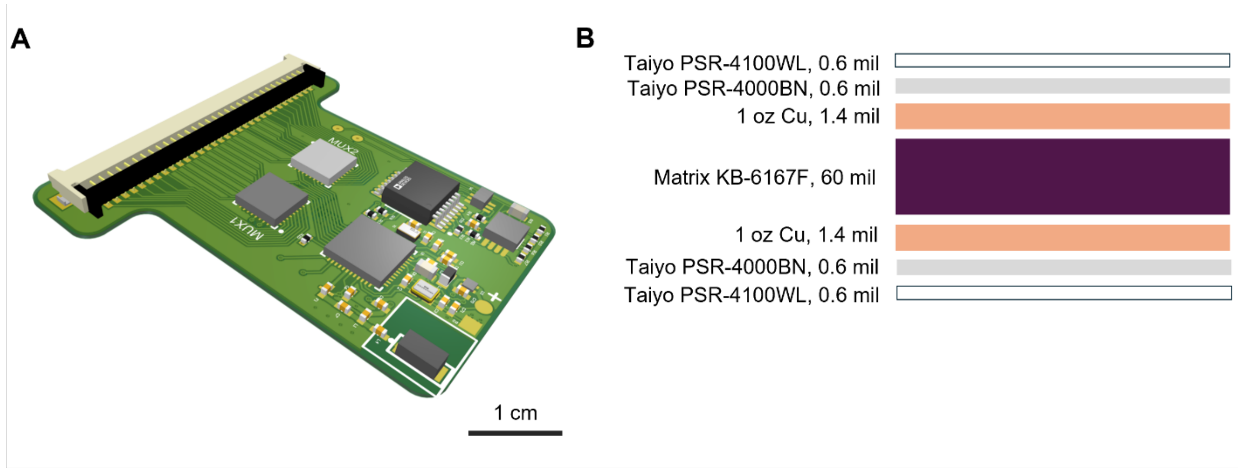

Figure S2. DAQ circuit. (A) 3D model of PCB design. (B) Schematic of stackup layers comprising PCB.

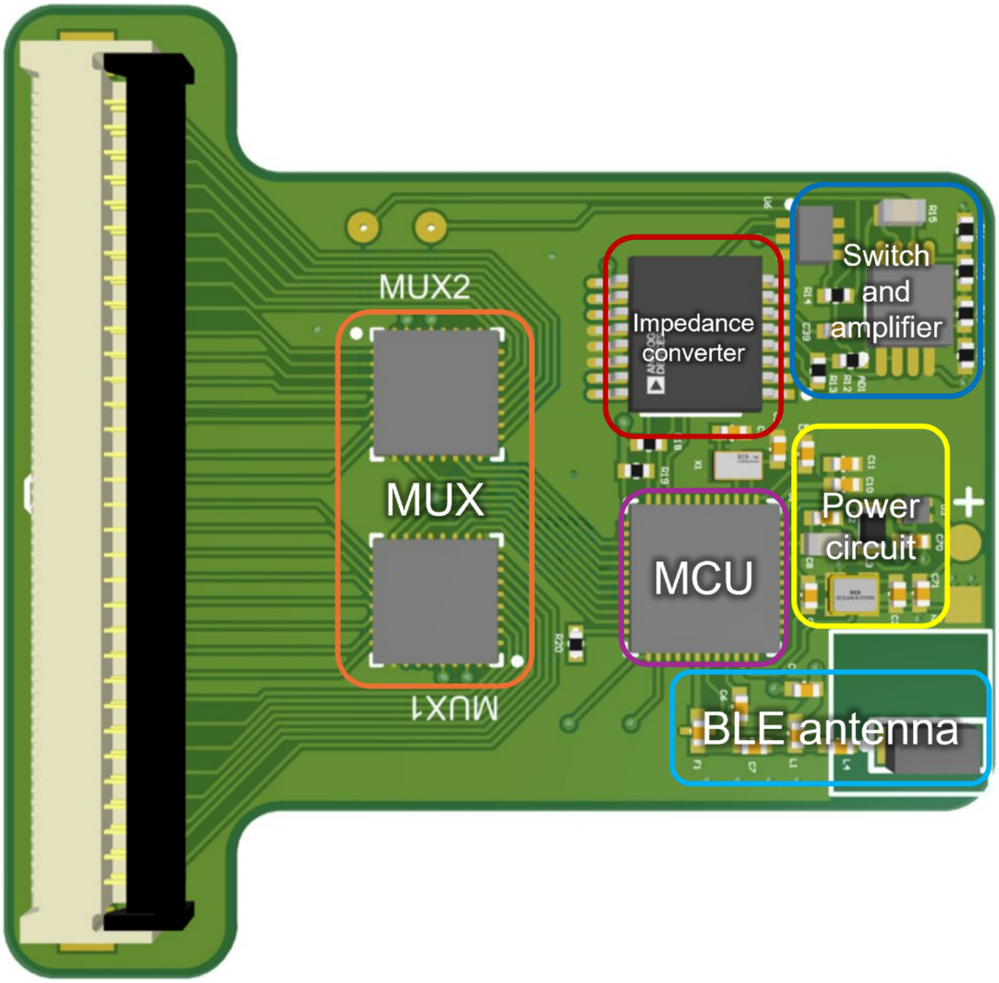

Component	Manufacturer	Purpose	Quantity
ADG1606	Analog Devices	Multiplexer	2
AD5933	Analog Devices	Impedance converter	1
NRF52832	Nordic Semiconductor	Microcontroller	1
ADG849	Analog Devices	Switch	1
AD8606	Analog Devices	Amplifier	1
XF3M (1)-3215-1B	Omron Electronics	FFC connector	1

Figure S3. Summary of IC components for 32-channel impedance DAQ circuit.

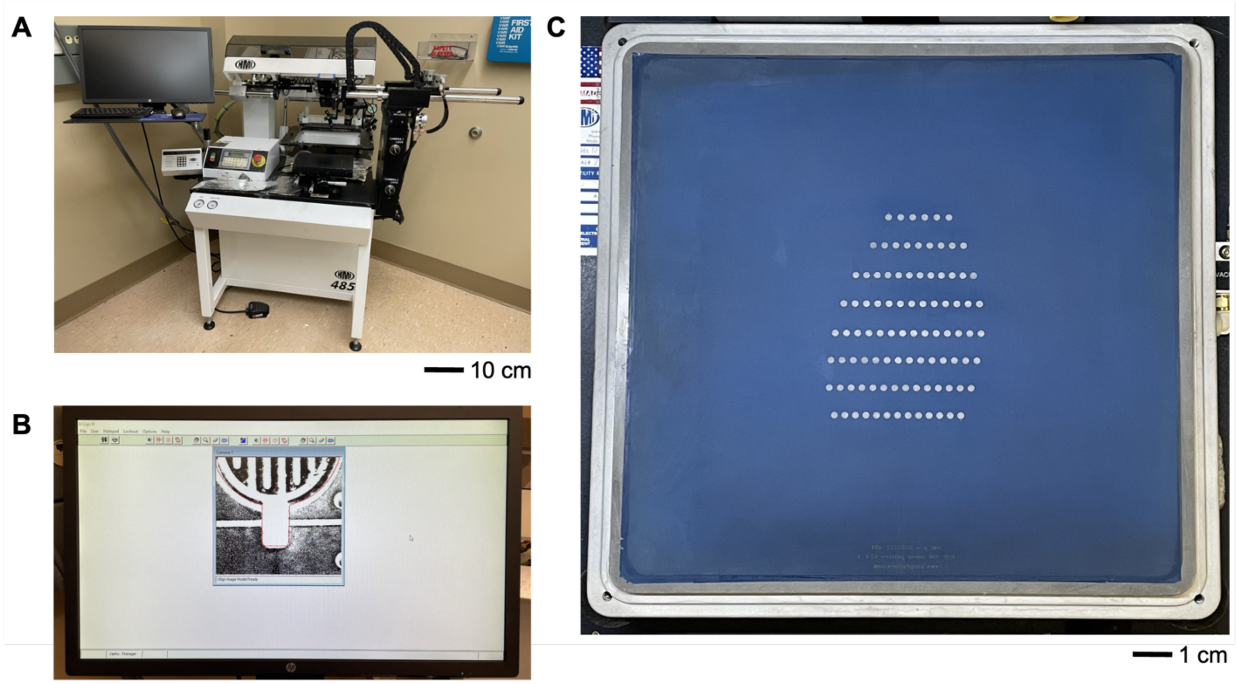

Figure S4. Screen-printing setup. (A) Hary Manufacturing Inc. MSP-485® screen printer. (B) Detail of laser-guided computer vision software used to align screen. (C) Screen for top half of insole.

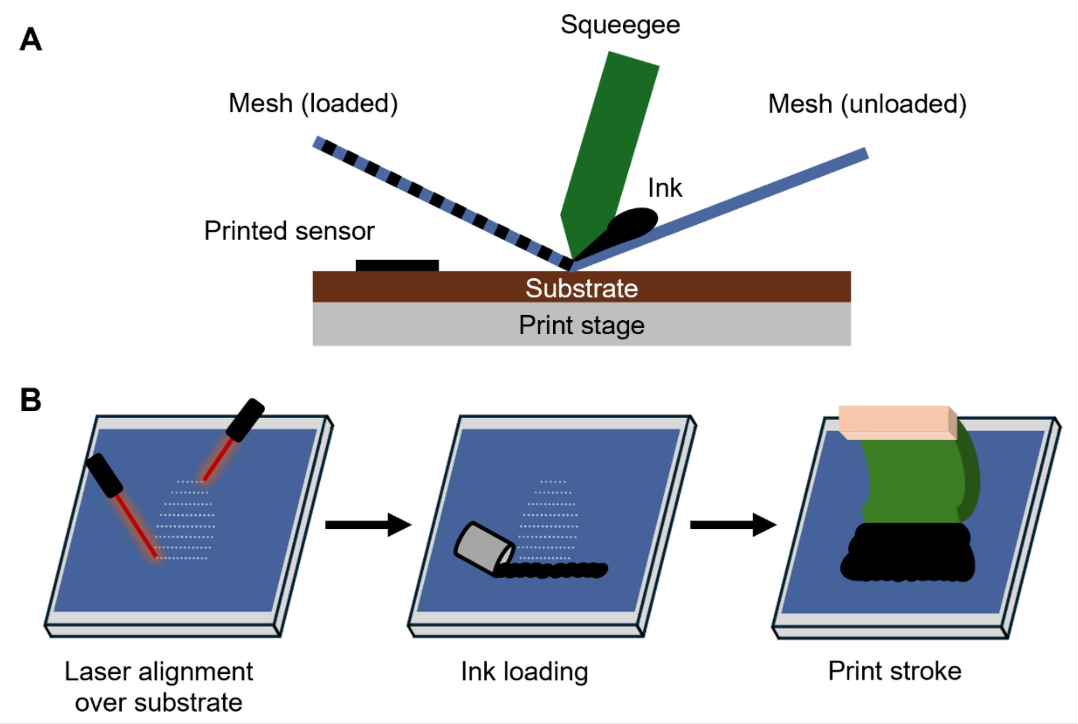

Figure S5. Screen-printing process. (A) Schematic of screen-printing mechanics. (B) Schematic of main steps in screen-printing process.

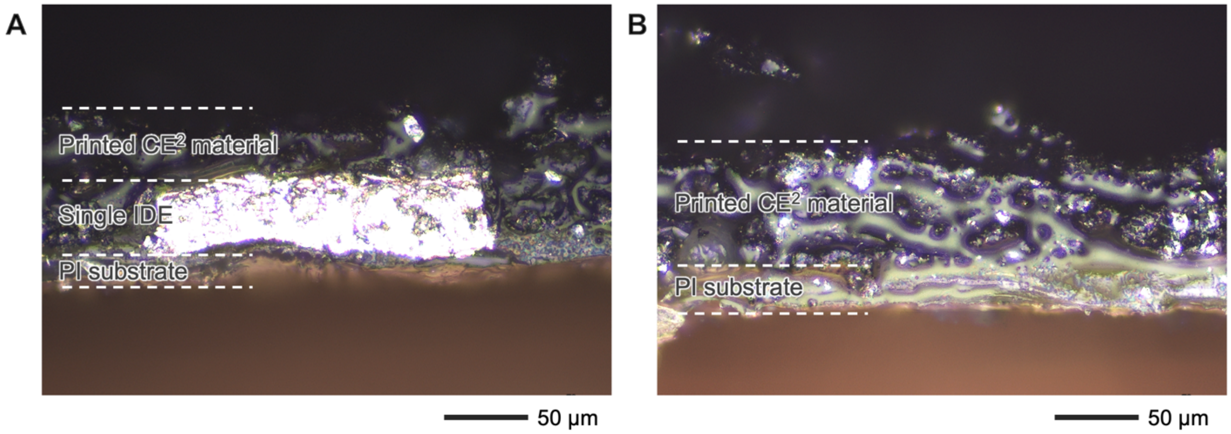

Figure S6. Cross-sectional view of the as-prepared sensor.

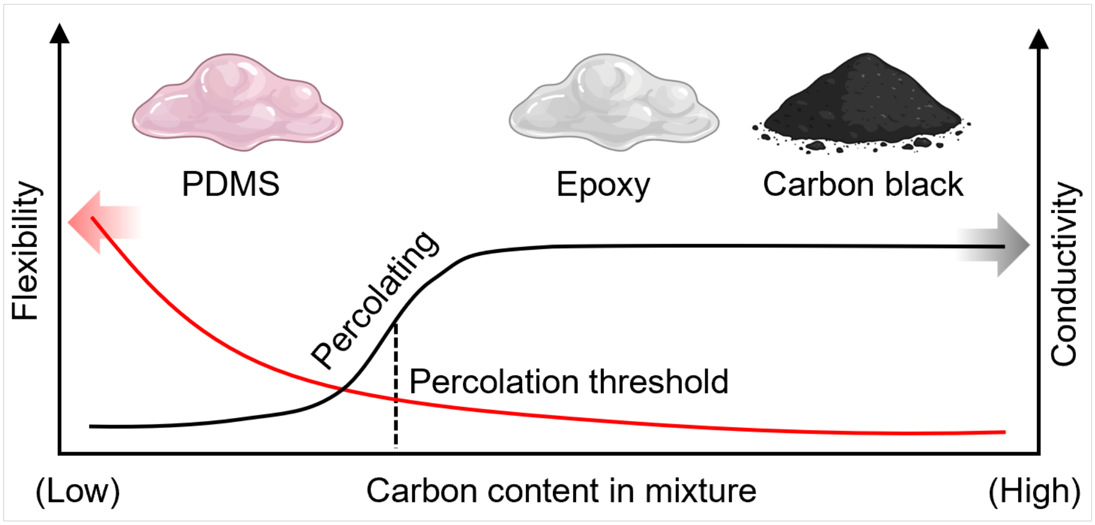

Figure S7. Relationship between sensor properties and carbon content. Major components of CE^2 ink mixture are shown.

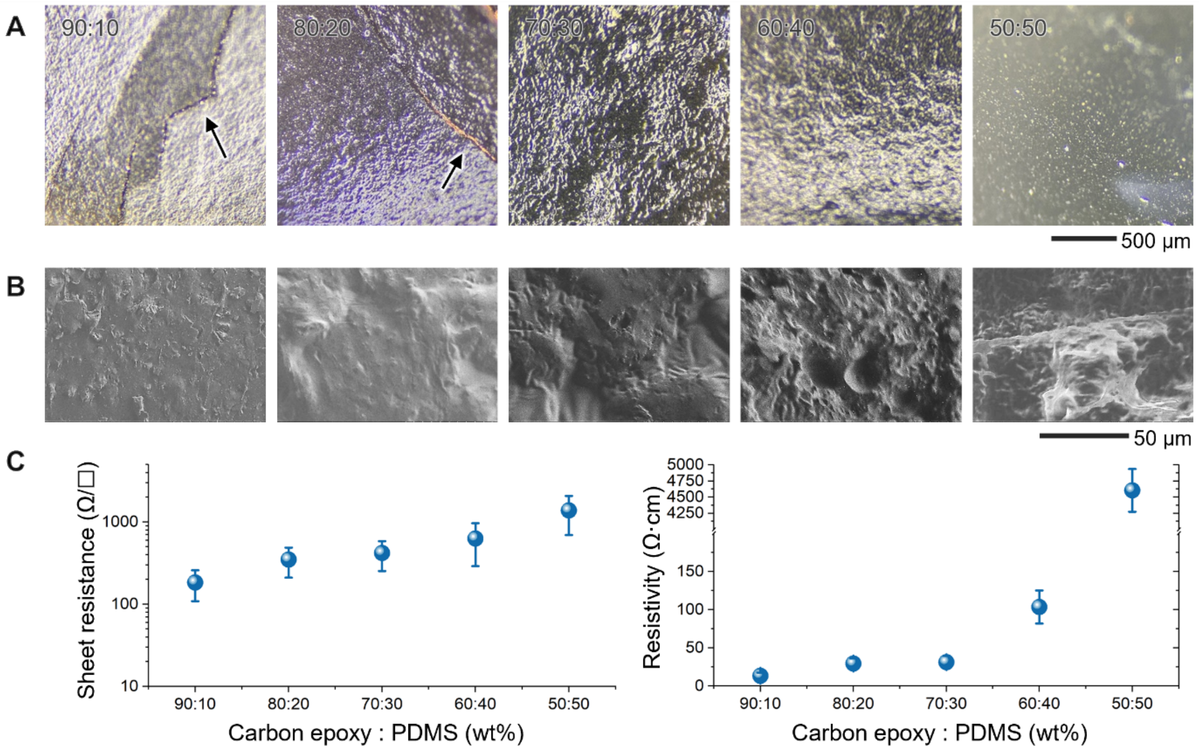

Figure S8. Surface profiles of printed samples with varying CE² ratios. (A) Optical micrographs of each sample (Carbon epoxy: PDMS, wt.%) show a crack in the 90:10 and 80:20 samples originating from poor flexibility. (B) FE-SEM micrograph views of the samples. (C) Sheet resistance and resistivity values for each sample.

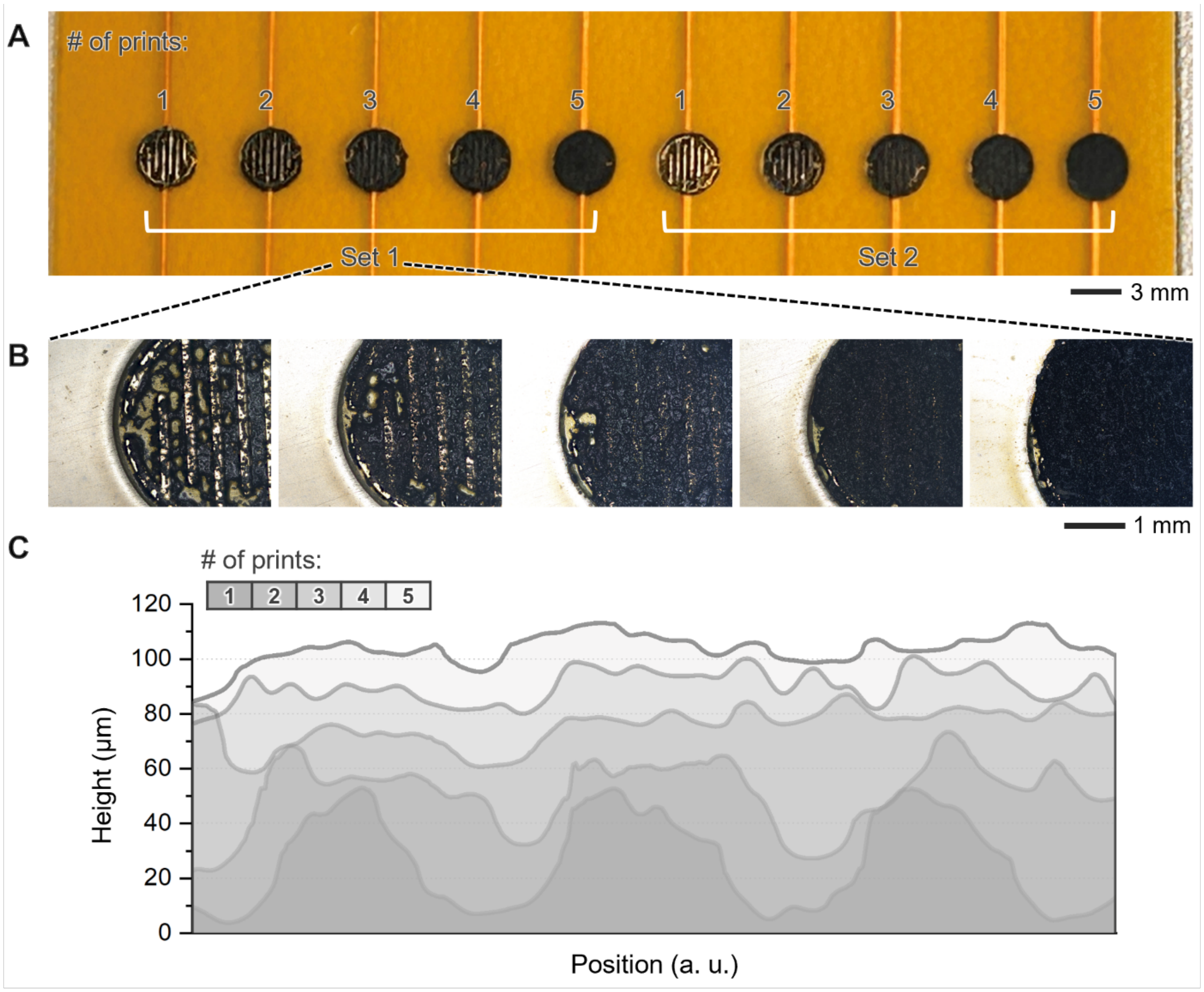

Figure S9. Surface profiles of printed samples with varying number of layers. (A, B) Optical views of each sample. (C) Height profiles with different numbers of printed layers.

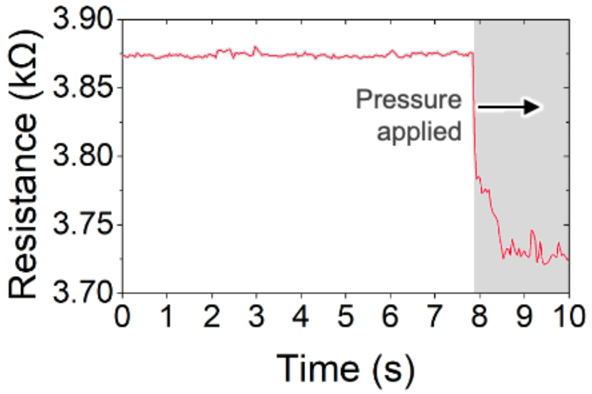

Figure S10. Characterization of sensor response time under applied pressure. A sudden drop in resistance is observed upon pressure application at ~8 s, indicating a rapid sensor response. The response time was measured to be approximately 72 ms, demonstrating the sensor's fast and reliable performance.

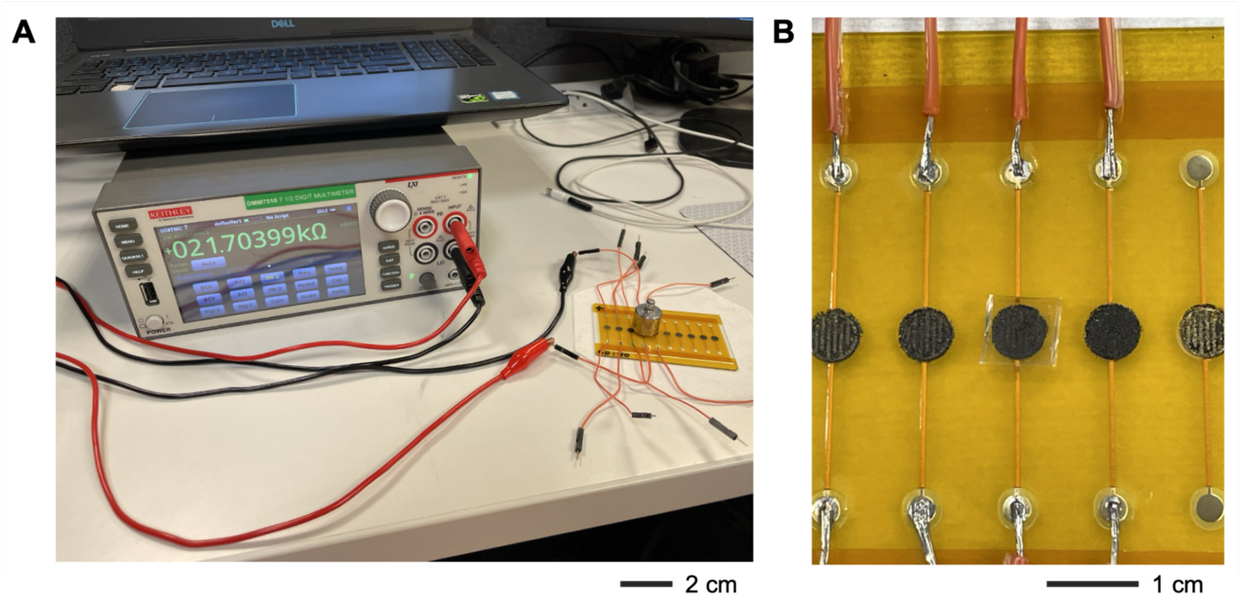

Figure S11. Test setup for sensor calibration. (A) IDEs connected to multimeter with calibration weight applied. (B) Detail of IDE with PDMS pad to control area over which pressure is applied.

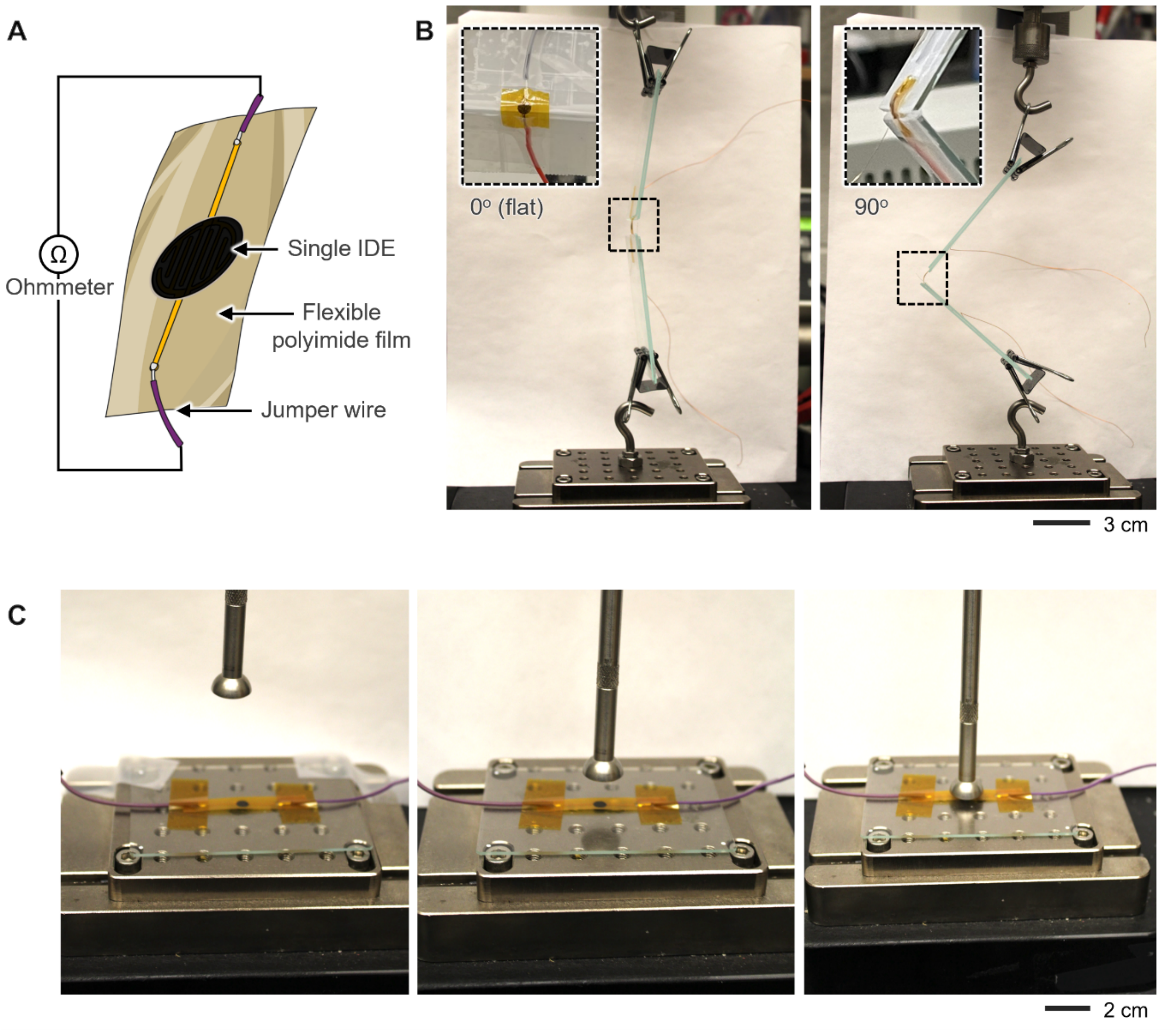

Figure S12. Test setup for durability study. (A) Schematic of specimen for testing. (B) Bending test. (C) Pressure response test.

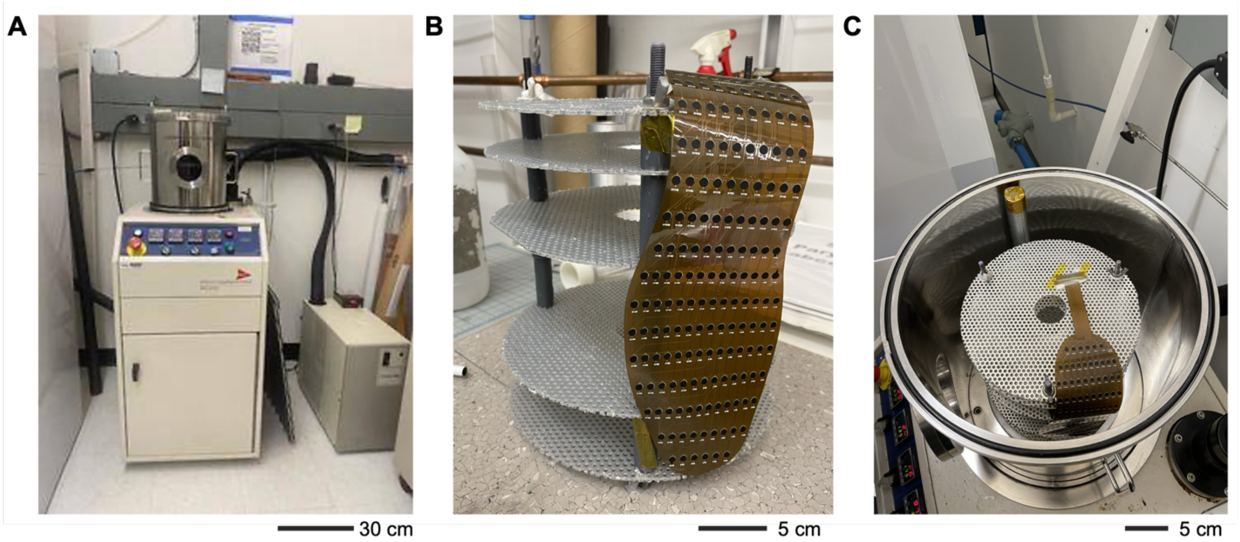

Figure S13. Parylene coating setup. (A) SCS Labcoter® parylene deposition system. (B) Insole secured to sample carrier using PI tape. (C) Carrier loaded into machine.
